# UAV-Assisted Cluster-Based Task Allocation for Mobile Crowdsensing in a Space–Air–Ground–Sea Integrated Network

**DOI:** 10.3390/s24010208

**Published:** 2023-12-29

**Authors:** Yang Liu, Yong Li, Wei Cheng, Weiguang Wang, Junhua Yang

**Affiliations:** 1 School of Electronics and Information, Northwestern Polytechnical University, Xi’an 710129, China; hnliuyang90@mail.nwpu.edu.cn (Y.L.);; 2 School of Information Engineering, Henan University of Science and Technology, Luoyang 471000, China; 3 School of Electronic Engineering, Xi’an University of Posts and Telecommunications, Xi’an 710121, China

**Keywords:** mobile crowdsensing, SAGSIN, edge computing, cluster algorithm, task allocation

## Abstract

Mobile crowdsensing (MCS), which is a grassroots sensing paradigm that utilizes the idea of crowdsourcing, has attracted the attention of academics. More and more researchers have devoted themselves to adopting MCS in space–air–ground–sea integrated networks (SAGSINs). Given the dynamics of the environmental conditions in SAGSINs and the uncertainty of the sensing capabilities of mobile people, the quality and coverage of the sensed data change periodically. To address this issue, we propose a novel UAV-assisted cluster-based task allocation (UCTA) algorithm for MCS in SAGSINs in a two-stage process. We first introduce the edge nodes and establish a three-layer hierarchical system with UAV-assistance, called “Platform–Edge Cluster–Participants”. Moreover, an edge-aided attribute-based cluster algorithm is designed, aiming at organizing tasks into clusters, which significantly diminishes both the communication overhead and computational complexity while enhancing the efficiency of task allocation. Subsequently, a greedy selection algorithm is proposed to select the final combination that performs the sensing task in each cluster. Extensive simulations are conducted comparing the developed algorithm with the other three benchmark algorithms, and the experimental results unequivocally endorse the superiority of our proposed UCTA algorithm.

## 1. Introduction

Over the past few years, with the explosive development of pervasive sensing and mobile communication technologies, Internet of Things (IoT) applications are no longer limited to exploring the land and sky but further extend to the perception, detection, and discovery of space and the sea. This trend has given rise to a new space–air–ground–sea integrated network (SAGSIN) with extraordinary capacities to comprehensively cover space, air, ground, and sea, which can aid in providing extremely wide coverage and ubiquitous flexible connection [1]. Generally speaking, SAGSINs usually leverage static wireless sensors, such as satellites, stationary sensors, cameras, buoys, and ships, to achieve the goal of pervasive sensing and cross-layer communication [2]. However, these traditional sensing methods are often limited due to high deployment and maintenance costs, and they cannot provide high spatiotemporal coverage due to insufficient sensors in certain areas, which may lead to quality loss in the sensing data [3] and has aroused widespread concerns about this important component of the future 6G network [4,5].

The proliferation and widespread use of mobile intelligent devices equipped with various types of built-in sensors (e.g., GPS, accelerometers, cameras, gyroscopes, etc.) motivate the emergence of a new sensing paradigm called mobile crowdsensing (MCS) [6]. Compared with the traditional sensing networks, MCS is a grassroots sensing paradigm that utilizes the idea of crowdsourcing [7] to outsource sensing tasks to ordinary mobile users with their omnipresent intelligent devices instead of stationary sensors, which can provide a guarantee for sensing and communication capacities [8]. In a typical MCS system, the cloud platform and mobile users are the most important components of the network. Upon receiving sensing tasks from the requesters or applications, the cloud platform publishes tasks and usually recruits mobile users to accomplish the tasks [9]. By making full use of the random mobility of mobile users, MCS allocates tasks to well-suited users, which can enhance the flexibility of ubiquitous sensing and ensure high spatiotemporal coverage. This appealing sensing paradigm, which can effectively achieve urban-scale monitoring, has expanded the scope of the IoT and has been widely used in many IoT applications, such as urban sensing [10,11], intelligent transportation [12,13,14], and environmental monitoring [15,16].

To overcome the restrictions of terrain and surfaces, and achieve a real sense of seamless global coverage, SAGSINs fully utilize traditional static sensing nodes such as satellites and pre-deployed stationary sensors while introducing new sensing methods to truly realize global ubiquitous connection [17]. Mobile crowdsensing, which applies the principles of crowdsourcing and takes advantage of the widespread distribution, flexible mobility, and the group intelligence of mobile people with powerful smart devices to perform urban-sensing tasks, has become the optimal method for SAGSINs to further improve their efficiency, especially in the air, ground, and sea fields [18]. Because of the advantages of MCS, more and more researchers have devoted themselves to adopting MCS in SAGSINs. Wang Y [17] introduced MCS into a SAGSIN to solve complex and large-scale sensing tasks and converted the traditional centralized application service architecture into a novel lightweight and distributed network architecture. Finally, they proposed a Q-learning-based task cooperation (QTC) mechanism based on edge computing, blockchain technology, and MCS. In order to conserve and utilize the oceans, seas, and their resources for sustainable development, Moroni D [19] presented an app based on a crowdsensing approach for SAGSINs to improve safety and awareness. However, although MCS is considered an effective scheme to meet several requirements of IoT applications, it still has many essential challenges to overcome, one of which is task allocation [20].

As a crucial stage of the MCS process, task allocation refers to how to appropriately assign different types of tasks to the eligible mobile users in MCS with the consideration of different factors, such as sensing coverage, budget constraint, energy consumption, and so forth [21]. Specifically, the cloud platform depends on a massive number of mobile users and recruits several of them to complete the tasks. Due to multifarious sensing positivity and the capacity of users for various tasks, if the tasks are randomly allocated to the users, the quality and credibility of the sensed data cannot be guaranteed, which could seriously affect the performance of the network [22]. Consequently, it is very important to design a reasonable task allocation strategy, which can significantly improve the accuracy and effectiveness of the MCS system. In recent years, studies of MCS task allocation have become a hotspot in academia, and many novel strategies have been proposed, with the purpose of maximizing sensing quality with a limited budget [23,24,25] or minimizing incentive costs while ensuring a minimal degree of sensing quality loss [26,27]. However, under the diversified dynamic MCS environment, particularly in SAGSINs, allocating large-scale tasks to mobile users according to heterogeneous sensors (e.g., mobile users, UAVs [28], ships, and stationary sensors) to achieve the tradeoff between the sensing quality and sensing cost is a huge challenge.

Because of the dynamics of the environmental conditions in SAGSINs and the uncertainty of the sensing capabilities of mobile people, the quality and coverage of the sensed data change periodically. In response to this challenge, to avoid the high task failure ratio, improve the sensing coverage quality, and reduce the sensing cost in dynamic urban scenarios, we concentrate on the MCS task allocation problem and propose a novel UAV-assisted cluster-based task allocation (UCTA) algorithm for mobile crowdsensing in SAGSINs to greatly increase the completion rate and achieve high coverage quality in multiple tasks within certain budgets. The main contributions of our work are as follows:

(1) Owing to the participants’ random mobility and uneven distribution in the urban environment, mobile crowdsensing (MCS) in SIGSIN encounters challenges characterized by heightened computational demands and prolonged task execution times. In response to this issue, we introduce the edge nodes and establish a three-layer hierarchical system with UAV assistance, called “Platform—Edge-Cluster—Participants”. This structure harnesses the computational capabilities and sufficient storage of edge nodes while enhancing the efficiency of task allocation and improving sensing data quality. Moreover, the task allocation problem is formulated to elevate the task completion rate and maximize sensing coverage under a certain budget.
(2) In order to generate an optimal task allocation plan, a novel UAV-assisted cluster-based task allocation for MCS in SAGSIN is proposed, which operates through a two-stage process. In the first stage, an edge-aided attribute-based cluster algorithm is designed, aiming at organizing tasks into clusters. This clustering process significantly diminishes both the communication overhead and computational complexity experienced by the cloud platform. Based on the attributes of the tasks and edge nodes, the cloud platform meticulously selects the suitable edge nodes as the cluster heads by comprehensively considering various factors of edge nodes, the working mode, computational power, and distance to form the cluster for each task. Subsequently, according to the expected rewards attribute of these IoT devices (participants, UAVs, and buoys), they will join the nearest cluster.
(3) Tailored to the different types of tasks encountered in urban scenarios, two distinct combinations are applied in the second stage to execute the tasks, ultimately enhancing task completion rates while maintaining the required sensing coverage and task quality. To select the final combination to perform the sensing task in each cluster, a greedy selection algorithm is proposed. This approach ensures the fulfillment of sensing coverage while operating within budget constraints, thereby striking a desirable balance between the expected rewards and budget limitations. Extensive simulations are conducted, comparing with the other three benchmark algorithms, and the experimental results unequivocally endorse the superiority of our proposed UCTA algorithm in terms of task completion rate and sensing coverage.

The remainder of our paper is organized as follows. In Section 2, we review and discuss the related works. The system model is expressed and the optimization problem is formulated in Section 3. To address the issue, we propose a novel UAV-assisted cluster-based task allocation (UCTA) algorithm for MCS in Section 4. Section 5 presents the simulation results compared with other algorithms. Finally, the conclusions about our paper are drawn in Section 6.

## 2. Related Work

In recent years, task allocation has grown to be a significant issue in MCS and has received significant scientific interest. More and more novel algorithms for task allocation in MCS have been presented, whose purposes generally aim to improve quality, reduce costs, and protect security and privacy. Reddy [29] took into account various factors, for example, users’ behaviors, time, and location restrictions, and proposed a coverage-based task allocation algorithm to choose the appropriate users in order to maximize the spatial coverage. Estrada, R [30] considered the time and location constraints and adopted some different mechanisms, such as the reputation mechanism, queuing mechanism, and principal mechanism, then designed a multi-task allocation strategy to reach the goal of revenue optimization. To overcome the problem of global optimization, Wang [31] focused on analyzing the problem of multi-task allocation and innovated the task-specific minimal sensing quality thresholds. Then, they proposed a novel multi-task allocation framework named MTasker to assign the different tasks to the users, which could maximize the overall system utility. In [32], Song investigated the misaligned task coverage problem caused by the popularity of tasks, which suggested that the popular tasks could attract sufficient users while less popular tasks may have few users to be assigned unsuccessfully. They combined the users’ task preferences and their attitudes on task attributes and designed a cTaskMat framework by utilizing the method of moving some certain qualified users to less popular tasks, to achieve the goal of increasing task coverage and reducing the costs.

As we know, MCS requires a large number of users to complete the tasks with high quality. However, in large-scale urban scenarios, there would be only a small number of users to participate in some areas, which could cause the failure of tasks. Recently, many studies have introduced unmanned aerial vehicles (UAVs) as a new exploration method that can bring a new dimension into MCS applications [33,34]. Because of the capacities of fast deployment and controllable mobility, a growing number of researchers expect that UAVs could replace human users to complete sensing tasks more efficiently and realize the autonomous MCS. Although UAVs can generally contribute sensing data more accurately and credibly than human users, there are many constraints about UAVs at present, for example, UAVs are forbidden in many cities. Hence, human users with mobile intelligent devices still play a significant role in MCS applications, while UAVs can act as the supplementary part to help human users accomplish sensing tasks accurately. H Gao [20] concentrated on UAV-assisted MCS applications and designed a UMA (UAV-assisted multi-task allocation method), which could maximize the sensing coverage and guarantee the data quality. B Wang [35] applied UAVs to disaster relief networks and proposed a social-aware UAV-assisted MCS system to, in stochastic and dynamic environments, recruit appropriate UAVs to replace human users to complete the tasks. They converted the task allocation problem into a dynamic matching problem and presented an MWTA algorithm in order to realize the optimal matching in a time-varying environment. In [36], L Fu investigated the problem of the failure of data collection in hard-to-reach and infrastructure-restrained urban areas and designed three-dimensional multi-UAV-assisted CrowdSensing, which was called 3DM. Compared with existing methods, 3DM fully utilized the 3D flexibility to enhance device matching and data transfer between UAVs and MDs, which could require less time and energy to accomplish the tasks.

Different from the existing works in the literature, our paper investigates the problem of task allocation for MCS in SAGSIN, which is relatively complicated in large-scale urban scenarios. The previous methods are no longer available in special scenarios. To address this issue, we introduce the edge nodes and establish a three-layer hierarchical system with UAV assistance, called “Platform—Edge-Cluster—Participants”. This structure harnesses the computational capabilities and sufficient storage of edge nodes while enhancing the flexibility and efficiency of task allocation and improving sensing data quality. Building on this foundation, a novel UAV-assisted cluster-based task allocation for MCS in SAGSIN is proposed, which operates through a two-stage process. UCTA utilizes the mobility of UAVs and ensures the fulfillment of sensing coverage while operating within budget constraints. This approach strikes a desirable balance between the expected rewards and budget limitations, offering a promising solution tailored to the distinctive demands of urban scenarios.

## 3. System Model and Problem Formulation

### 3.1. System Model

**Figure 1:**
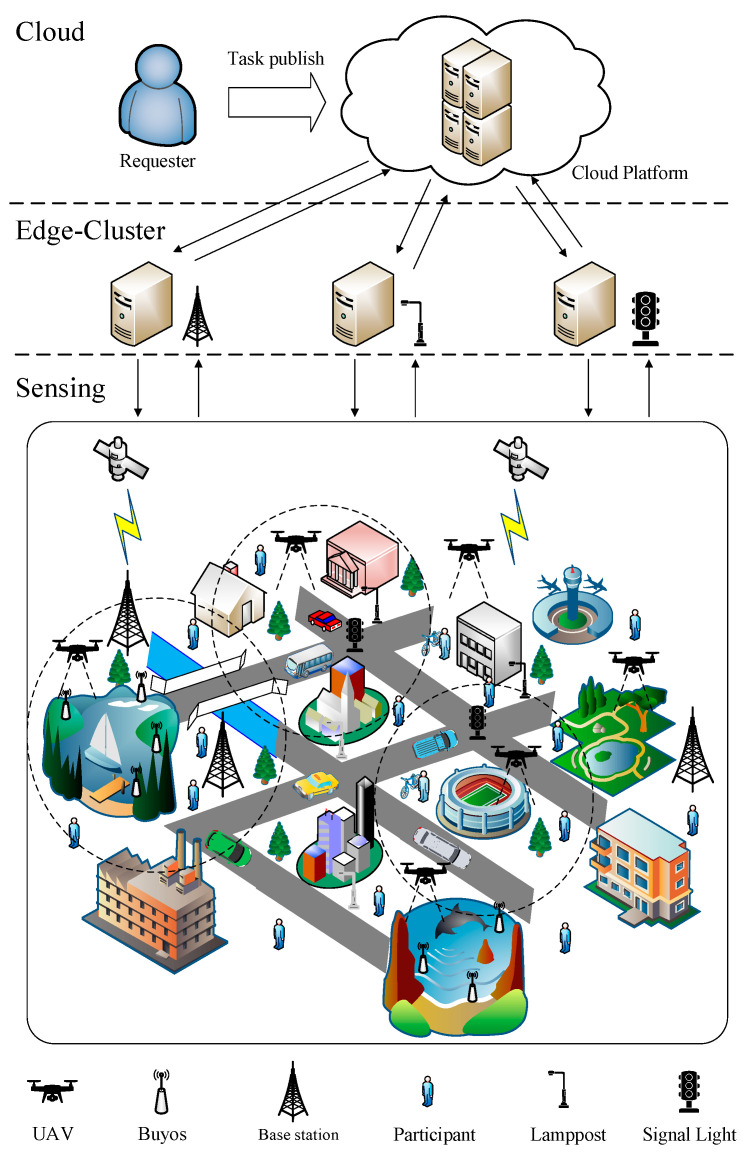
“Platform—Edge-Cluster—Participants” MCS system.

We consider that the MCS system works in urban environments. As illustrated by Figure 1, similar to the typical MCS system, we assume that the requestors or applications send requirements to the cloud platform P, which provides services for smart cities every day. Then, the cloud platform P publishes a set of concurrent sensing tasks $\theta_{total}$ denoted by $\theta_{total}=\left(\theta_{1},\theta_{2},\ldots ,\theta_{j}\right)$. Each task has certain sensing types and different demands, which can be described extensively as a multi-element tuple $F_{\theta }=\left(\theta_{j}^{typ},l_{\theta_{j}},t_{\theta_{j}},A_{\theta_{j}},CP_{\theta_{j}},B_{\theta_{j}}\right)$, where $\theta_{j}^{typ}$ indicates the sensing type of task θ. In our system, there are two different sensing types $\theta_{j}^{typ}=\left(\theta_{L-A}^{typ},\theta_{W-A}^{typ}\right)$, which include land-air mode and water-air mode. The land-air mode task $\theta_{L-A}^{typ}$ intends to acquire the data of the urban land region, and the water-air mode task $\theta_{W-A}^{typ}$ aims to obtain data for urban lake or ocean regions. The UAVs will assist the participants in completing these tasks. The rest of the elements represent the coordinates of the location, time limitations, sensing coverage requirements, required computational power, and certain task budget, respectively. Additionally, to better satisfy the time constraints of the task, the entire sensing campaign is usually divided into K timeslots with the same length.

We assume that there are a certain number of participants $U=\left(u_{1},u_{2},\ldots ,u_{U}\right)$ who are willing to take part in and accomplish the tasks. For any participant $u_{i}$ who wants to engage in the tasks, the participant will upload his/her detailed information to the cloud platform P at the beginning of the tasks. The relevant attributes of the participant $U=\left(u_{1},u_{2},\ldots ,u_{U}\right)$ can be represented as Fu=lui,SAui,Reuiθj, where $l_{u_{i}}$ denotes the real-time location information of the participant $u_{i}$, $SA_{u_{i}}$ represents the sensing coverage ability of the participant $u_{i}$, which refers to sensing radius in meters, and Reuiθj is the expected reward for the participant $u_{i}$ to accomplish the task $\theta_{j}$. Meanwhile, there is a set of UAVs $V=\left(v_{1},v_{2},\ldots ,v_{n}\right)$ cruising in the sky of the city and a group of static nodes, such as smart lampposts, base stations, and especially sensorized buoys $B^{S}=\left(b^{S}{}_{1},b^{S}{}_{2},\ldots ,b^{S}{}_{n}\right)$, pre-deployed in the lake or ocean to sense the water data. As we know, due to the uncertainty of the data quality sensed by the participants and limitations of the sensing range of mobile devices, the UAVs and sensorized buoys will be a perfect complement for different task types.

In order to serve as a bridge to connect the cloud platform P and the participants, an edge-cluster layer is introduced into our MCS system, which can preprocess the participants’ information more closely and tackle the problems caused by different types of tasks more effectively. We assume that a set of edge nodes $EN=\left(EN_{1},EN_{2},\ldots ,EN_{l}\right)$ is pre-deployed in the city and the attribute of EN can be denoted as $F_{EN}=\left(M_{EN},l_{EN},CP_{EN}\right)$, where $M_{EN}$ is the mode of the edge nodes. $M_{EN}=1$ is working mode, and $M_{EN}=0$ is sleep mode. $l_{EN}$ and $CP_{EN}$ represent the location and computing power. Accordingly, the workflow of our MCS system can be described as follows. The cloud platform P publishes the tasks $\theta_{total}=\left(\theta_{1},\theta_{2},\ldots ,\theta_{j}\right)$ each round from the requestors or applications. Then, the edge-cluster layer collects and handles the participants’ information who are willing to finish the tasks. Based on the different types and requirements of tasks, the edge layer helps the cloud platform P assign tasks to the most appropriate participant or static node. The UAVs will function as a supplement to accomplish the tasks.

### 3.2. Problem Formulation

For the two types of tasks, we consider that the cloud platform P will assign tasks to different combinations to complete the tasks. In the land-air mode, the task can be assigned to a participant with a mobile device and a UAV to achieve optimal coverage and acquire better-quality data. Meanwhile, in the water-air mode, given that the participants are unable to collect the data on water more effectively, the sensorized buoy and a UAV can replace the participant to accomplish the tasks. Because the sensorized buoys $B^{S}=\left(b^{S}{}_{1},b^{S}{}_{2},\ldots ,b^{S}{}_{n}\right)$ with built-in sensors are stationary and pre-deployed in the water regions, we assume that the sensorized buoy $B^{S}$, whose attributes can be denoted as $F_{B^{S}}=\left(SA^{B^{S}},C_{\theta_{j}}^{B^{S}}\right)$, can accomplish the task $\theta_{j}$. The sensing coverage ability of $B^{S}$ is $SA^{B^{S}}$, and the energy cost is $C_{\theta_{j}}^{B^{S}}$. In our system, the UAVs can fly around the whole city and sense the data. The detailed attributes of the UAVs $V=\left(v_{1},v_{2},\ldots ,v_{n}\right)$ can be denoted as $F_{V_{A}}=\left(SA^{V_{A}},C_{\theta_{j}}^{V_{A}}\right)$, where $SA^{V_{A}}$ is the sensing coverage ability of the UAV, and $C_{\theta_{j}}^{V_{A}}$ represents the cost for the task $\theta_{j}$. For simplicity, we think the $C_{\theta_{j}}^{V_{A}}$ is directly related to the flying distance and proportional to it [19], i.e., $C_{\theta_{j}}^{V_{A}}=\gamma d_{\theta_{j}}^{V_{A}}$, where $d_{\theta_{j}}^{V_{A}}$ denotes the flying distance for the sensing task $\theta_{j}$.

In our paper, the goal is to maximize the sensing coverage quality and improve the completion rate of the two types of tasks with budget and energy constraints in urban scenarios. Therefore, we introduce the equation named coverage-completed ratio (CCR) [20] to reveal the ratio of the sensed coverage area and the required coverage area. The objective function can be expressed as

(1) $$\begin{array}{ll} \max CCR & =\sum_{l=1}^{L}\sum_{j=1}^{J}\frac{A_{\theta_{j}}^{SA}}{A_{\theta_{j}}^{RA}}\\ & =\sum_{l=1}^{L}\sum_{j=1}^{J}\frac{\left|A_{\theta_{m},L-A}^{SA}\right|+\left|A_{\theta n,W-A}^{SA}\right|}{A_{\theta_{j}}^{RA}}\\ & =\sum_{l=1}^{L}\sum_{j=1}^{J}\frac{\left|A_{\theta_{m}}^{P}\underset{m\in \left\{1,2,\ldots ,M\right\}}{\cup }A_{\theta_{m}}^{V}\right|+\left|A_{\theta n}^{B^{S}}\underset{n\in \left\{1,2,\ldots ,N\right\}}{\cup }A_{\theta n}^{V}\right|}{A_{\theta_{j}}^{RA}} \end{array}$$

(2) s.t.m+n=j,ReL−Aθm≤Bθm,ReW−Aθn≤Bθn,∑j=1JReθj≤Btotal,

where L denotes the rounds of tasks. m and n denote the numbers of the two types of tasks, respectively. $A_{\theta_{j}}^{SA}$ and $A_{\theta_{j}}^{RA}$ represent the sensed coverage area and the required coverage area. $\left|A_{\theta_{m},L-A}^{SA}\right|$ and $\left|A_{\theta n,W-A}^{SA}\right|$ are the sensed coverage area of land-air mode tasks and water-air mode tasks. $\left|A_{\theta_{m}}^{P}\underset{m\in \left\{1,2,\ldots ,M\right\}}{\cup }A_{\theta_{m}}^{V}\right|$ and $\left|A_{\theta n}^{B^{S}}\underset{n\in \left\{1,2,\ldots ,N\right\}}{\cup }A_{\theta n}^{V}\right|$ are the effective coverage area for two types of tasks, respectively. In addition, the CCR (Equation (1)) should satisfy these constraints (2) simultaneously. The total rewards should be less than or equal to the maximum budgets of whole tasks.

We can see that Equation (1) is a combinatorial optimization problem. In [37], we know that the task allocation in MCS is proven to be an NP-hard problem. It is difficult to find the optimal solution in polynomial time. The key of the CCR equation is to maximize the sensed coverage area for each task $\theta_{j}$ under either type in whole rounds. Consequently, we can convert Equation (1) into the new function, expressed as

(3) maxAθjSAs.t.m+n=j,  ReL−Aθm≤Bθm,  ReW−Aθn≤Bθn.

For each task $\theta_{j}$, no matter whether a land-air mode task or water-air mode task, when the required coverage area is fixed, the goal is converted to find the maximum sensed coverage area and satisfy the constraints of the budget and energy. We need to find the optimal combination to maximize the sensed coverage area of land-air mode tasks and water-air mode tasks $\left|A_{\theta_{m},L-A}^{SA}\right|$ and $\left|A_{\theta n,W-A}^{SA}\right|$. And the reward Reθj for each task $\theta_{j}$ should be less than or equal to the limited budget of the task $\theta_{j}$. Then, for the whole sensing tasks $\theta_{total}=\left(\theta_{1},\theta_{2},\ldots ,\theta_{j}\right)$, we can obtain a collection of the combinations under budget constraints to achieve the maximum sensing coverage area for each task $\theta_{j}$.

## 4. UAV-Assisted Cluster-Based Task Allocation Algorithm for MCS in SAGSIN

In this section, we propose a novel UAV-assisted cluster-based task allocation for MCS in SAGSIN in a two-stage process. In the first stage, an edge-aided attribute-based cluster algorithm is introduced to help tasks form the clusters, which can significantly reduce the communication traffic and computational complexity for the cloud platform. In the second stage, a greedy selection algorithm is presented to choose the appropriate combination to allocate and finish the tasks. The key symbols and notations are summarized in Table 1.

### 4.1. Edge-Aided Attribute-Based Cluster Algorithm

In the traditional MCS system, it usually operates in a “Platform—Participants” model. The cloud platform directly publishes and assigns the tasks to participants, and then the participants sense, collect, and transfer the data to the cloud platform, which may lead to some shortcomings. Although the mobile participants are the most important component of the MCS system, they are full of uncertainty. Due to the participants’ random mobility and uneven distribution in the urban environment, this will cause chaos and confusion in the MCS system, which increases the computational load and the time consumption [38]. The emergence of UAVs and static nodes further raises the computational burden and communication energy consumption for the cloud platform. In light of this, inspired by the clustering mechanism, we introduce the edge nodes and establish a three-layer hierarchical system, called “Platform—Edge-Cluster—Participants”, like in Figure 1, which can leverage the edge nodes with excellent computing capabilities and sufficient storage, for instance, smart lampposts [39,40], base stations [41], and signal light [42]. These edge nodes can preprocess and compute the sensing data at the edge of the network, which can significantly reduce the communication traffic and computational complexity, further improving the task allocation and the sensing quality effectively. However, how to select a suitable edge node as the cluster head to group the participants, UAVs, and buoys will be a great challenge.

Consequently, we propose an edge-aided attribute-based cluster algorithm, and the specific process is shown in Algorithm 1. Upon acquiring the tasks $\theta_{total}=\left(\theta_{1},\theta_{2},\ldots ,\theta_{j}\right)$ from the requestors or applications, the cloud platform will allocate the tasks based on attributes of tasks to the edge nodes and choose the most suitable edge node to assign the task to participants, UAVs, and buoys. In one duration, an edge node only executes one task. The number of published tasks in each time slot determines the number of clusters. For each cluster CL, the location of the tasks will be regarded as the center of the clusters. Based on the attributes $F_{EN}=\left(M_{EN},l_{EN},CP_{EN}\right)$ of these edge nodes EN, the cloud platform first gathers the attributes of EN and confirms that each edge node is in the working mode $M_{EN}$. Next, the cloud platform calculates the distance information between each EN and task, which can be denoted as

(4) $$Dist\left(EN_{l},\theta_{j}\right)=\sqrt{{\left(x_{EN_{l}}-x_{\theta_{j}}\right)}^{2}+{\left(y_{EN_{l}}-y_{\theta_{j}}\right)}^{2}},$$

where $\left(x_{EN_{l}},y_{EN_{l}}\right)$ is the location attributes of the edge nodes $EN_{l}$, and $\left(x_{\theta_{j}},y_{\theta_{j}}\right)$ is the latitude and longitude of the task $\theta_{j}$. The task’s proximity to the edge node directly correlates with its efficiency in task completion. In addition, the tasks also have the requirements of computational power, which means that EN should have adequate computing power to carry the computational load. Therefore, we can attain the relationship between EN and cloud platform as

(5) $$CP_{\theta_{j}}\leq CP_{EN_{l}}.$$

According to these factors, the cloud platform chooses the optimal edge node for each task $\theta_{j}$ as the cluster head.
**Algorithm 1.** Edge-Aided Attribute-Based Cluster Algorithm**Input:** input θ, UV, $B^{S}$, EN
**Output:** $CL=\left(CL_{1},CL_{2},\ldots ,CL_{l}\right)$
  1: Calculate all $Dist\left(EN_{l},\theta_{j}\right)$
  2: **for** l=1 to j **do**
  3:     **if** $M_{EN}$ $=1,\;CP_{\theta_{j}}\leq CP_{EN_{l}}$$,\;\min\;Dist\left(EN_{l},\theta_{j}\right)$   4:     $EN_{l}$ become cluster head for $CL_{l}$  5:    updates the set of clusters CL
  6: **end for**
  7: Calculate all $Dist\left(u_{i},\theta_{j}\right)$,$Dist\left(v_{n},\theta_{j}\right)$,$Dist\left(b^{S}{}_{n},\theta_{j}\right)$
  8: **for** i=1 u, **do**
  9:     **if** min $Dist\left(u_{i},\theta_{j}\right)$, Reuiθj<Bθj
 10:     $u_{i}$ joins the cluster $CL_{l}$
 11:    **end if**
 12: **end for**
 13: **for** m=1, v=1 **to** n, **do**

 14:    **if** min $Dist\left(v_{n},\theta_{j}\right)$$,C_{\theta_{j}}^{V_{A}}<B_{\theta_{j}}$
 15:     $v_{n}$ joins the cluster $CL_{l}$
 16:    **if** min $Dist\left(b^{S}{}_{n},\theta_{j}\right)$$,C_{\theta_{j}}^{B^{S}}<B_{\theta_{j}}$
 17.     $b^{S}{}_{n}$ joins the cluster $CL_{l}$
 18:    **end if**
 19: **end for**
 20: **Return** all cluster $CL=\left(CL_{1},CL_{2},\ldots ,CL_{l}\right)$


Subsequently, as mentioned above, in land-air mode, the edge nodes group the participants and UAVs into clusters according to their attributes as candidates, such as locations, energy, reward, etc. In the water-air mode, the edge nodes operate in the same way to cluster the sensorized buoys and UAVs. Hence, the distance between the IoT devices (participants, UAVs, and buoys) and the tasks occupies a vital position in forming a cluster. Specifically, the edge nodes need to calculate the distance between these IoT devices and tasks based on their location attributes. Since the participants usually roam in the city and have to travel through the blocks to execute the tasks, the Manhattan distance [43] is applied to calculate the distance between the participants and the tasks, which can be expressed as

(6) $$Dist\left(u_{i},\theta_{j}\right)=\left|x_{u_{i}}-x_{\theta_{j}}\right|+\left|y_{u_{i}}-y_{\theta_{j}}\right|,$$

where $\left(x_{u_{i}},y_{u_{i}}\right)$ denotes the latitude and longitude of the participant. On the contrary, UAVs and buoys are cruising in the sky of the city or are stationary in th e water region. Therefore, we simply use the Euclidean distance to compute the distance between them and the tasks, which can be expressed as

(7) $$Dist\left(v_{n},\theta_{j}\right)=\sqrt{{\left(x_{v_{n}}-x_{\theta_{j}}\right)}^{2}+{\left(y_{v_{n}}-y_{\theta_{j}}\right)}^{2}},$$

(8) $$Dist\left(b^{S}{}_{n},\theta_{j}\right)=\sqrt{{\left(x_{b^{S}{}_{n}}-x_{\theta_{j}}\right)}^{2}+{\left(y_{b^{S}{}_{n}}-y_{\theta_{j}}\right)}^{2}},$$

where $\left(x_{v_{n}},x_{v_{n}}\right)$ and $\left(x_{b^{S}{}_{n}},y_{b^{S}{}_{n}}\right)$ denote the location attributes of the UAV $v_{n}$ and buoy $b^{S}{}_{n}$. Based on the distance information, the participants, UAVs, and buoys will join the cluster where the task is closest to them, which ensures all these IoT devices will be in a cluster and be the candidates for the tasks. Furthermore, the budget is also a constraint to consider for the edge node to absorb the participants, UAVs, and buoys to form the cluster, which should be satisfied as

(9) Reuiθj<BθjCθjVA<Bθj.CθjBS<Bθj

Combining all these factors, the clusters $CL=\left(CL_{1},CL_{2},\ldots ,CL_{l}\right)$ for the tasks are finalized.

### 4.2. Greedy Selection Algorithm

After the edge nodes EN have formed the clusters $CL=\left(CL_{1},CL_{2},\ldots ,CL_{l}\right)$ for each task $\theta_{j}$ and all these IoT devices (participants, UAVs, and buoys) join each closest cluster, the task $\theta_{j}$ selects these devices in the cluster as the optimal candidates to execute tasks. The problem can be transformed into how to allocate each task $\theta_{j}$ to the proper participant or buoy with the UAV assistance in the cluster, aiming to achieve a sensing coverage area as large as possible without violating the budget specified by the tasks. The edge nodes EN only need to choose the appropriate combination (one participant with a UAV, or one buoy with a UAV) in the clusters to accomplish the tasks, which greatly reduces the computational loads and improves the overall system efficiency at the same time.

According to the analysis above, in the dynamic urban scenarios, for different types $\theta_{j}^{typ}=\left(\theta_{L-A}^{typ},\theta_{W-A}^{typ}\right)$ of tasks, we assume that two combinations are applied to execute the tasks, which can significantly increase the completion rate of tasks while providing assurance of the sensing coverage and the quality of tasks. In land-air mode, these sensing tasks always need to be executed in the urban land area, such as traffic monitoring, noise conditions, and air conditions, where a large number of people with mobile devices can be the perfect carrier to sense and collect the data. Meanwhile, the UAV-assist solution can help people complete tasks more accurately and quickly. However, in water-air mode, some tasks should be sensed over the water or ocean, which is not convenient for people to sense and collect the required data. These sensorized buoys $B^{S}=\left(b^{S}{}_{1},b^{S}{}_{2},\ldots ,b^{S}{}_{n}\right)$ and UAVs provide excellent chances for the remote evaluation of several elements, such as water quality and vessel traffic at different scales, from worldwide coverage data to the detailed assessment of small urban areas.

For the purpose of improving the completion rate and maximizing the sensing coverage quality, while reducing the cost, we propose a greedy selection algorithm to help each cluster choose the optimal combination to assign the task, which can finish the task quickly, obtain the best possible coverage in terms of quality, but demand less reward for their costs. No matter whether in land-air mode or water-air mode, the sensing coverage ability, expected reward of participants, and the costs of UAVs and buoys play a vital role in selection. Considering all these factors, we built the fitness function, which can be expressed as:

(10) f=αAθjSAAθjRA+(1−α)1−ReθjBθj,

where the first part represents the sensing coverage ability of these IoT devices (participant, UAV, and buoy), while the second part indicates that the less reward they demand, the greater the possibility they will be selected. And α is the balancing parameter, ranging from 0 to 1. The fitness function can provide a guarantee for sensing coverage in both land and water regions under the relatively lower budget constraints, which effectively achieves a better tradeoff between the reward and the budget. Algorithm 2 presents more details about the greedy selection algorithm.
**Algorithm 2.** Greedy Selection Algorithm**Input:** input $CL=\left(CL_{1},CL_{2},\ldots ,CL_{l}\right)$,θ, U, V, $B^{S}$
**Output:** the set of selected participants, UAVs and buoys ${Χ}^{*}$
  1: **for** $CL_{l}$ **in** CL **do**
  2:  **for** $u_{i}$,$v_{n}$,$b^{S}{}_{n}$ in $CL_{l}$ **do**
  3:    Calculate all f scores
  4:    Sort by descending order
  5:    select the max f scores of combination $x^{*}$
  6:   **end for**
  7: **end for**
  8:  ${Χ}^{*}={Χ}^{*}\cup \left(x^{*}\right)$
  9: **Return** ${Χ}^{*}$


### 4.3. Process of UAV-Assisted Cluster-Based Task Allocation Algorithm

**Figure 2:**
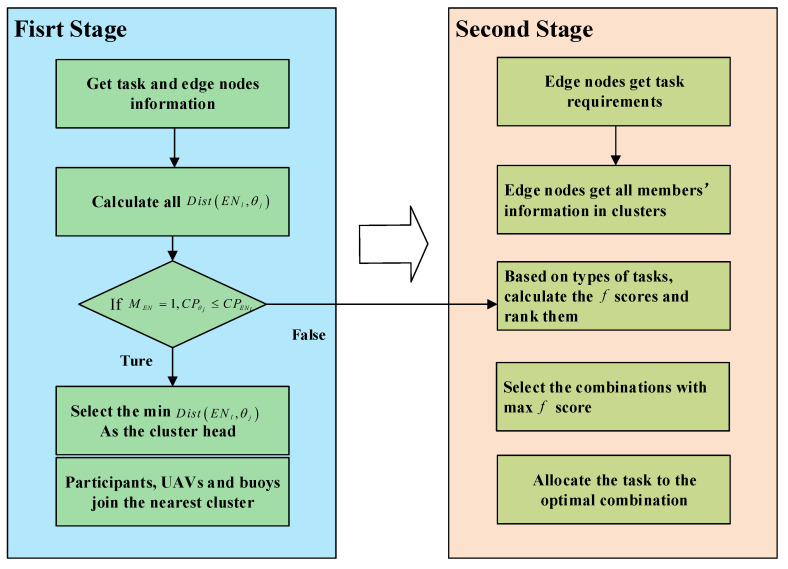
Flowchart of UCTA algorithm.

To help the cloud platform allocate the tasks to the suitable participants, UAVs and buoys, we analyze the coverage problem about MCS, then design a novel UAV-assisted cluster-based task allocation algorithm for MCS in SAGSIN, which includes two stages. The specific process of this task allocation algorithm is presented as follows and shown in Figure 2.

**First Stage:** Based on the different locations of the tasks, we introduce the edge-cluster mechanism, which can assist edge nodes in choosing proper candidates to form the cluster for each task. In the beginning, comprehensively considering various factors of edge nodes, such as the working mode, computational power, and distance between the edge nodes and tasks, the cloud platform chooses the optimal edge node as the cluster head for each task. Afterward, according to the expected reward attributes of these IoT devices (participants, UAVs, and buoys), they will join the nearest cluster. After these IoT devices that are eligible for the requirements are added to each cluster, the set of clusters $CL=\left(CL_{1},CL_{2},\ldots ,CL_{l}\right)$ for the tasks is finalized.

**Second Stage:** For different types $\theta_{j}^{typ}=\left(\theta_{L-A}^{typ},\theta_{W-A}^{typ}\right)$ of tasks, we assume that two combinations are applied to execute the tasks, which can significantly increase the completion rate of tasks while providing assurance of the sensing coverage and the quality of tasks. We leverage a greedy selection algorithm to select the final combination (participants with UAV, or buoy with UAV), which performs the sensing task in each cluster, which can provide the guarantee for sensing coverage under relatively lower budget constraints and effectively achieve a better tradeoff between the reward and the budget.

## 5. Simulation and Numerical Results

In this section, we conduct extensive simulations and evaluate the performance of our proposed UAV-assisted cluster-based task allocation (UCTA) algorithm for MCS in SAGSIN. To better validate the analysis, we compare the UCTA algorithm with the other three benchmark baselines, the UMA [20], PETA [44], and GA-TA [45] algorithms, in terms of task completion rate and task coverage rate. The UMA method concentrates on the MCS situations with UAV assistance and designs a task allocation method, aiming at acquiring the maximum sensing coverage and data quality. The PETA algorithm utilizes the edge nodes to cluster and manage users and proposes a privacy-preserving edge task allocation method for MCS. The GA-TA algorithm considers the task allocation problem for only UAVs and proposes a genetic algorithm-based task allocation algorithm to minimize the sensing cost and ensure the quality of data. Then, we discuss the results to demonstrate the advantages of our UCTA algorithm.

Considering the simulation scenario, changing variables and parameters from other references, our simulations are performed by a discrete-event simulator designed for research use in mobile crowdsensing, which is called CrowdSenSim [46]. It enables the simulation of large-scale crowdsensing operations in realistic urban environments and can be applied to develop creative solutions for task allocation, user selection, resource management, and monitoring. In our simulation, we set the simulation area in the real city of Venice in Italy. We assume that the participants with mobile devices and UAVs are randomly distributed throughout the city, where the participants roam along the roads at a constant velocity, and UAVs can fly to a destination at a faster speed. The edge nodes and buoys are pre-deployed all over the city and water regions. According to [45,47], Table 2 summarizes the detailed experimental settings in our simulation.

**Figure 3:**
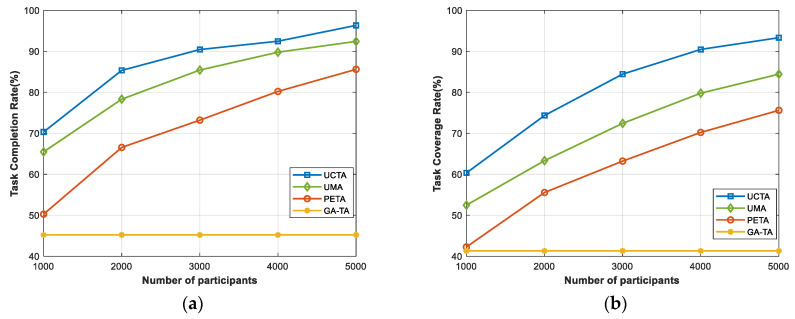
Performances under various numbers of participants. (**a**) Task completion rate; (**b**) task coverage rate.

In Figure 3, when we change the number of participants, we try to compare the performance in terms of task completion rate and task coverage rate between UCTA and the other three algorithms. It is easily seen that as the number of participants who are willing to take part in and execute the tasks continues to increase from 1000 to 5000, the curves present an upward trend in two indicators, except for the GA-TA algorithm, which strongly indicates that the quantity of participants plays a significant role in execution for MCS applications. The GA-TA algorithm only recruits the UAVs to accomplish the tasks; however, the participants are the essential components for the other three algorithms. The enlarged number of participants means that there are more candidates for each task to choose from, which will generate an excellent task allocation plan. From Figure 3a, when the number of participants equals 5000, with the UAV assistance, our proposed UCTA algorithm can reach a task completion rate of 96%, which outperforms the other two baseline methods. As depicted in Figure 3b, the task coverage rate using our UCTA is substantially improved compared with UMA and PETA.

**Figure 4:**
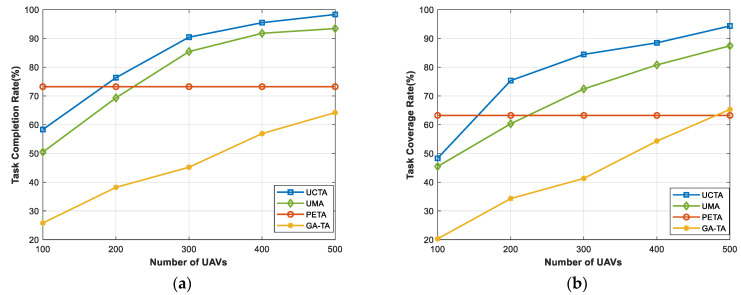
Performances under various numbers of UAVs. (**a**) Task completion rate; (**b**) task coverage rate.

Figure 4 shows the performances under an increasing number of UAVs. Clearly, since the PETA algorithm does not consider the UAVs to complete the tasks, the curve remains constant in both task completion rate and task coverage rate. However, our proposed UCTA and other benchmarks have a continuously improving performance with the rise in the number of UAVs. Obviously, from the figure, when the number of UAVs is set to 100, the performances of the task completion rate and task coverage rate are maintained at a relatively low level. This is because the number of UAVs is too small, which means that there are not adequate combinations to complete the tasks and attain high-quality sensing coverage. But, as the number of UAVs rises, there will be more and more suitable UAVs out there to choose from to accomplish the tasks, meanwhile guaranteeing the sensing coverage. As expected, the results keep on rising, and when the number of UAVs equals 500, our UCTA can achieve almost 98% task completion rate and 95% task coverage rate, which are clearly superior to other benchmark algorithms.

**Figure 5:**
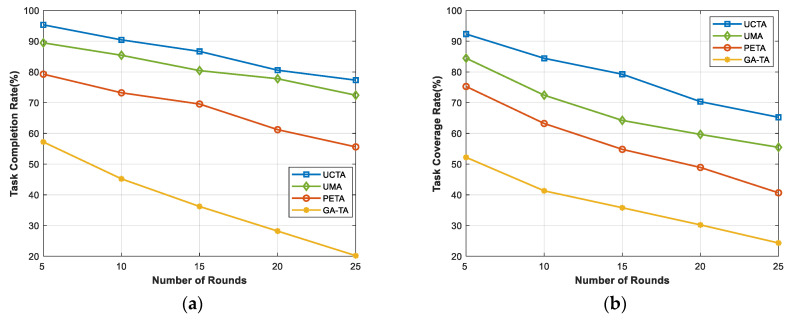
Performances under different rounds. (**a**) Task completion rate; (**b**) task coverage rate.

In Figure 5, we evaluate the performances of task completion rate and task coverage rate under different rounds. It is clear that the results demonstrate a declining trend. This is because, with more rounds, the number of tasks will be growing and there will be insufficient participants and UAVs to accomplish the tasks. If more tasks are completed, these IoT devices (participants, UAVs, and buoys) will consume more energy. As the rounds progress, they will not be able to finish more tasks. As depicted in Figure 5a, when the rounds equal 5, our UCTA can achieve a 96% task completion rate and outperform the other three benchmark algorithms. Even at 25 rounds, UCTA still can obtain a nearly 80% task completion rate. The GA-TA method is the most affected by the increase in rounds. The reason is that UAVs cost more energy to execute tasks. If they cannot be charged, they will run out of energy fast. Likewise, the mobile devices of the participants will lose power quickly. Similar to Figure 5a,b illustrates that our UCTA can achieve more extensive sensing coverage than UMA, PETA, and GA-TA methods.

**Figure 6:**
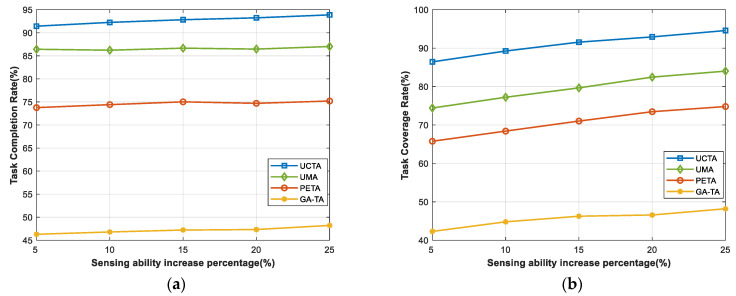
Performances under sensing ability increased. (**a**) Task completion rate; (**b**) task coverage rate.

In Figure 6, we try to verify the relationship between the performance of the task completion rate and task coverage rate with the sensing ability of these IoT devices (participants, UAVs, and buoys) increasing. It can be seen from Figure 6a that when the whole sensing ability increases, the task completion rate grows slightly and tends to be flat. The reason is that, although the sensing ability of devices is increasing from 5% to 25%, this does not determine whether the tasks can be completed. It always depends on whether there are sufficient participants, UAVs, or buoys to execute the tasks. On the contrary, Figure 6b displays a growing trend in the task coverage rate. The sensing ability of devices is related to whether they can obtain more sensing coverage, which will greatly and effectively improve the performance of the task coverage rate. The results in Figure 6b are consistent with our expectations that our UCTA algorithm takes a huge advantage and leads by at least 12% ahead of the other three benchmark algorithms.

**Figure 7:**
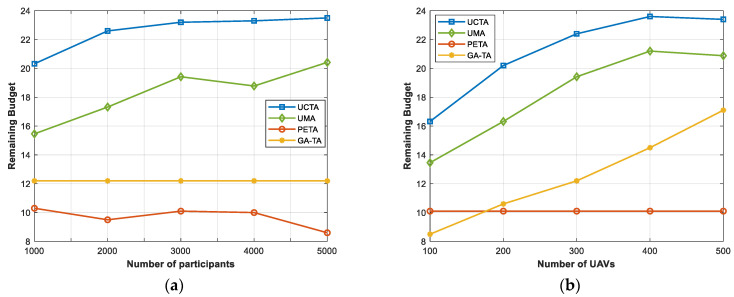
Remaining budget under various numbers of (**a**) participants; (**b**) UAVs.

In Figure 7, we investigate the impacts of the number of participants and UAVs on the remaining budget. As we know, in the case of a constant number of tasks, the total budget is limited. The cloud platform should select the proper participants or UAVs under the budget constraint, which may result in failure to generate the optimal plan. However, with the number of participants and UAVs increasing, there will be more possibilities and choices so that the cloud platform utilizes the limited budget to select more suitable participants or UAVs to complete the tasks. The results in Figure 7 reveal that our UCTA is outstanding compared to the other three algorithms under the varying numbers of participants and UAVs, which is consistent with our goal of improving the completion rate and maximizing the sensing coverage quality while reducing the cost.

## 6. Conclusions

The proliferation and widespread use of mobile intelligent devices equipped with various types of built-in sensors (e.g., GPS, accelerometers, cameras, gyroscopes, etc.) motivate the emergence of a new sensing paradigm, mobile crowdsensing (MCS). MCS takes advantage of the widespread distribution, flexible mobility, and the group intelligence of mobile people with powerful smart devices to perform urban-sensing tasks, which has been the optimal method for SAGSIN to further improve efficiency, especially in the air, ground, and sea fields. However, how to apply MCS to complex urban scenario applications in SAGSIN has raised huge challenges. Hence, designing a reasonable task allocation algorithm for MCS to address the various issues in SAGSIN has become increasingly crucial and urgent. In our work, we propose a novel UAV-assisted cluster-based task allocation (UCTA) algorithm for mobile crowdsensing in SAGSIN to greatly increase the completion rate and achieve the high coverage quality of multiple tasks under certain budgets. Firstly, we introduce the edge nodes and establish a three-layer hierarchical system with UAV assistance, called “Platform—Edge-Cluster—Participants”. This structure harnesses the computational capabilities and sufficient storage of edge nodes while enhancing the flexibility and efficiency of task allocation and improving sensing data quality. An edge-aided attribute-based cluster algorithm is designed to organize tasks into clusters. Building on this foundation, a greedy selection algorithm is proposed to select the final combination, which performs the sensing task in each cluster. This approach strikes a desirable balance between the expected rewards and budget limitations, offering a promising solution tailored to the distinctive demands of urban scenarios. Extensive simulations are conducted to compare with the other three benchmark algorithms, UMA, PETA, and GA-TA. The experimental results unequivocally endorse the superiority of our proposed UCTA algorithm.

## Figures and Tables

**Table 1:** Symbols and notations.

Symbols and Notations	Explanation
θ, U, V, $B^{S}$, EN	Set of tasks, participants, UAVs, buoys, and edge nodes
$F_{\theta }$, $F_{u}$, $F_{V_{A}}$, $F_{B^{S}}$, $F_{EN}$	Attributes of tasks, participants, UAVs, buoys, and edge nodes
$\theta_{j}^{typ},l_{\theta_{j}},t_{\theta_{j}},A_{\theta_{j}},CP_{\theta_{j}},B_{\theta_{j}}$	Type, location, time limitation, sensing coverage requirements, required computational power, and budget of tasks
lui,SAui,Reuiθj	real-time location, sensing coverage ability, and the expected reward of participants
$M_{EN},l_{EN},CP_{EN}$	Mode, location and computing power of the edge nodes
$SA^{V_{A}},C_{\theta_{j}}^{V_{A}}$	sensing coverage ability and cost of the UAVs
$SA^{B^{S}},C_{\theta_{j}}^{B^{S}}$	sensing coverage ability and cost of the buoys
$A_{\theta_{j}}^{SA}$, $A_{\theta_{j}}^{RA}$	the sensed coverage area and the required coverage area for task $\theta_{j}$

**Table 2:** Simulation settings.

Parameters	Value
Number of tasks each round	50
Number of participants	3000
Number of UAVs	300
Number of buoys	50
Number of edge nodes	100
Computing power of the edge nodes	[10, 20]
Budget	[100, 200]
Coverage ability of participants	[10, 50]
Coverage ability of UAVs	[25, 80]
Coverage ability of buoys	[50, 100]
Evaluation period	8:00 a.m.–18:00 p.m.
Task duration	20 min
Timeslot duration	2 min
Rounds	10
balancing coefficient α	0.7, 0.3

## Data Availability

Data are contained within the article.
